# Modeling personalized heart rate response to exercise and environmental factors with wearables data

**DOI:** 10.1038/s41746-023-00926-4

**Published:** 2023-11-15

**Authors:** Achille Nazaret, Sana Tonekaboni, Gregory Darnell, Shirley You Ren, Guillermo Sapiro, Andrew C. Miller

**Affiliations:** 1 Columbia University, New York, NY USA; 2 University of Toronto, Toronto, ON Canada; 3 Apple, Cupertino, CA USA

**Keywords:** Machine learning, Data integration, Biomarkers, Cardiovascular biology

## Abstract

Heart rate (HR) response to workout intensity reflects fitness and cardiorespiratory health. Physiological models have been developed to describe such heart rate dynamics and characterize cardiorespiratory fitness. However, these models have been limited to small studies in controlled lab environments and are challenging to apply to noisy—but ubiquitous—data from wearables. We propose a hybrid approach that combines a physiological model with flexible neural network components to learn a personalized, multidimensional representation of fitness. The physiological model describes the evolution of heart rate during exercise using ordinary differential equations (ODEs). ODE parameters are dynamically derived via a neural network connecting personalized representations to external environmental factors, from area topography to weather and instantaneous workout intensity. Our approach efficiently fits the hybrid model to a large set of 270,707 workouts collected from wearables of 7465 users from the Apple Heart and Movement Study. The resulting model produces fitness representations that accurately predict full HR response to exercise intensity in future workouts, with a per-workout median error of 6.1 BPM [4.4–8.8 IQR]. We further demonstrate that the learned representations correlate with traditional metrics of cardiorespiratory fitness, such as VO_2_ max (explained variance 0.81 ± 0.003). Lastly, we illustrate how our model is naturally interpretable and explicitly describes the effects of environmental factors such as temperature and humidity on heart rate, e.g., high temperatures can increase heart rate by 10%. Combining physiological ODEs with flexible neural networks can yield interpretable, robust, and expressive models for health applications.

## Introduction

The increasing availability of wearable technologies has empowered individuals to monitor their overall health and well-being throughout daily life—a recent research study found that 21% of Americans use wearable fitness trackers or smartwatches^1^. Such wearable data carry the potential for machine learning (ML) models to discover new correlates of human health from device signals^2^. Previous work has shown success in various applications, from clinical monitoring tools to fitness and activity planners^3^. In the clinical domain, modern ML methods have been shown to predict cardiovascular events from wearables data^4–6^, as well as overall health conditions indicated by different lab measures^7^. In the public health domain, wearables data and ML algorithms have been successfully deployed in disease surveillance^8^ such as detecting influenza-like illness^9^, monitoring disease progression in the population^10,11^, and informing the design of new epidemiological studies^12^. This relatively recent area of research demonstrates that wearables data is a rich source of information that can provide insight into an individual’s overall health.

Heart rate response to activity or exercise reflects an individual’s cardiorespiratory fitness. In sports medicine, exercise stress testing is used to quantify cardiac health by measuring cardiac response to a controlled physical activity^13^. Physiological models of heart rate response to exercise intensity have been developed to measure cardiorespiratory in controlled settings in small studies^14–17^. Similar principles may be used to monitor cardiorespiratory health throughout daily activities using wearables data, as opposed to controlled-environment tests. However, there are two major challenges. First, these physiological models of heart rate response must be adapted to use data collected by wearables—e.g., step count, speed, elevation change, and weather. Second, personalizing these models to measure an individual’s cardiorespiratory fitness is both computationally and statistically challenging. Given the prevalence of wearables, such health monitoring could reach a broader population with an increased frequency, and without interruption to daily life.

In this work, we develop a scalable algorithm that predicts heart rate response to workout intensity as measured by wearables—step count, speed, and elevation change. We augment an expert model from the exercise physiology literature with machine learning components and inference techniques^14^. Our approach combines a physiological model of heart rate (HR) based on ordinary differential equations (ODEs) with neural networks and representation learning to estimate personalized, user-specific parameters. Our algorithm learns to map a subject’s recent workout history to a personalized representation that is predictive of HR response in future workouts under the ODE model. This is done with data from the Apple Heart and Movement Study (AHMS)^18^, rendering this work as one of the largest and in regular environments (outside of the lab) reported in the literature on this topic.

We show that our personalized representations and model can accurately estimate the heart rate profile given workout data sequences by simply using an individual’s workout history. Unlike most existing work in the literature that performs short-term HR prediction^19,20^, our method can predict the entire HR trend of a completely new workout of up to 2 h. These learned health representations of individuals could be used in a variety of applications, such as personalized workout planning and estimating HR zones or calories burned during a workout.

The ability to accurately estimate HR response to any workout shows that the representations summarize meaningful information about an individual’s health. We investigate this by showing that, after being learned only for predicting heart rate, the learned representations correlate well with traditional metrics of cardiorespiratory fitness, e.g., VO_2_ max. Additionally, we explicitly show evidence of an effect of weather on heart rate during workouts across the study population; this is done by augmenting the biophysical ODE mode to consider environmental factors.

## Methods

### Study design and participants

Our study uses workout measurements contributed to the Apple Heart and Movement Study (AHMS) between November 2019 and July 2022^18^. The Apple Heart and Movement Study is a prospective, single-group, open-label, siteless, pragmatic observational study conducted in collaboration with the American Heart Association and Brigham and Women’s Hospital to investigate the relationship between physical activity, mobility, and heart health. The study was approved by the Advarra Central Institutional Review Board (PRO00036784) and registered to ClinicalTrials.gov (ClinicalTrials.gov Identifier: NCT04198194). There is no compensation for participation.

The AHMS allows participants to securely share information collected by their Apple Watch. To be eligible, the participants must own an Apple Watch paired with their iPhone, be at least 18 years old (at least 19 years old in Alabama and Nebraska; at least 21 years old in Puerto Rico), live in the United States of America, have installed the Apple Research app on their iPhone, do not share their iCloud account, iPhone, or Apple Watch with anyone else, and are willing and able to provide informed consent to participate in the study. The study app was used to verify eligibility, obtain participants’ consent, provide study education, and direct participants through the study procedures.

**Fig. 1:**
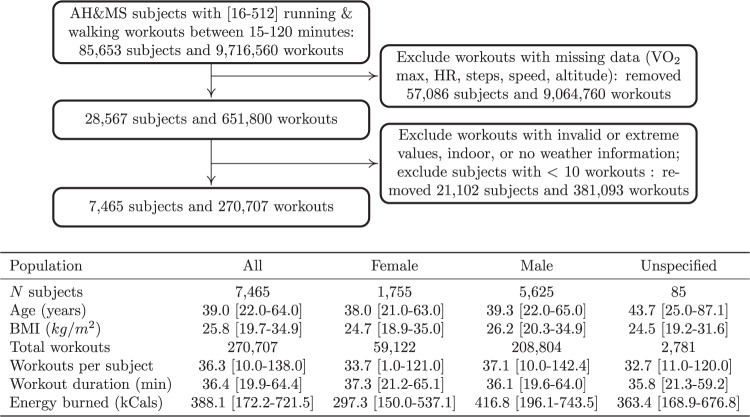
Cohort summary. **a** Subject and workout inclusion diagram. **b** Description of study data and summary statistics of included participants. Intervals reflect the 2.5 and 97.5 quantile range.

Within the AHMS, we selected participants who chose to use the Workout app on their Apple Watch to record their outdoor runs. The AHMS logs the outdoor weather information *W* (temperature and humidity) at the time of each workout and provides the participant’s heart rate during each workout along with four measurements of exercise intensity: the instantaneous speed from the pedometer sensor, the instantaneous speed from the GPS, the step cadence from the pedometer, and the elevation gain from the altimeter. The sensor measurements are interpolated on a 10-s grid to form, for each workout *w*, a heart rate time-series $\widehat{{{{\rm{HR}}}}}\in {{\mathbb{R}}}^{d}$ and a multivariate time-series of exercise intensity $I\in {{\mathbb{R}}}^{4\times d}$; where *d* is the duration of the workout. We finally only selected workouts with a duration between 15 and 120 min long that had no missing data and further filtered out participants with less than 10 valid runs. We obtained 270,707 outdoor runs from 7465 distinct subjects (see Fig. 1 for cohort summary and inclusion criteria).

**Fig. 2:**
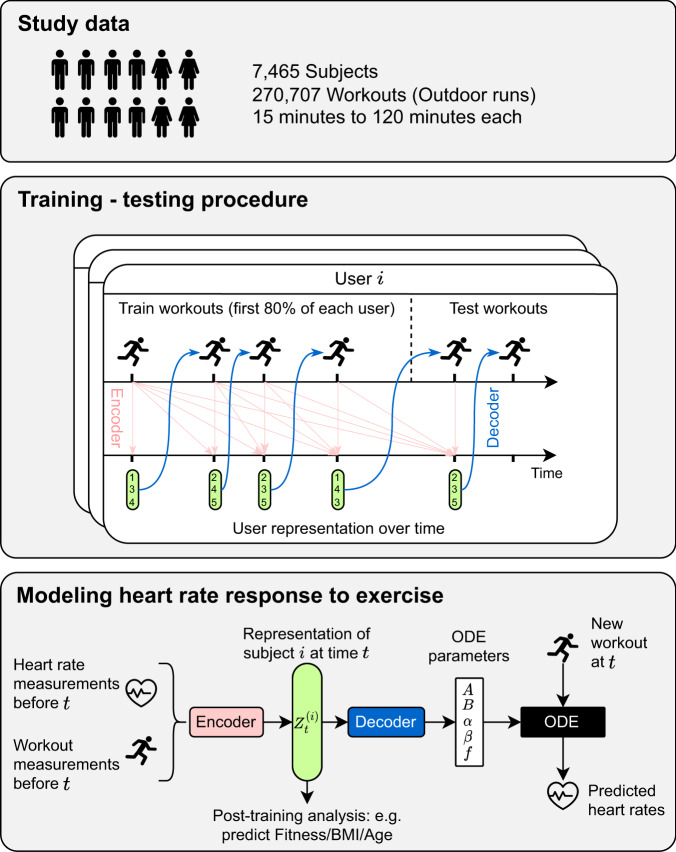
Method overview. Overview of the method for modeling heart rate response to exercise with wearable data. The top panel describes the study population data and details of the wearable workout measurements used. The two bottom panels describe the training procedure and how the model components are trained to learn the personalized representation and ODE parameters using only heart rate and workout data. Learned representations can then be used downstream to predict various physiological traits, e.g., Fitness, BMI, and age.

Our hybrid approach blends a physiological model of heart rate dynamics with machine learning components (e.g., deep neural networks) to adapt it to wearable data and personalize it to individual subjects. We first detail the physiological model and then describe our ML-based augmentations and learning algorithm. A diagram of the end-to-end system is detailed in Fig. 2.

### Physiological HR dynamics model

Several works in the sports physiology literature have studied heart rate dynamics in response to exercise using ordinary differential equations (ODEs)^14–17^. These approaches translate the physical mechanisms of the human body into differential equations in order to incorporate domain (physiology) knowledge in the modeling. This is an appealing method to build interpretable and ultimately trustworthy models of fitness and health.

A common approach for modeling changes in heart rate, HR, due to exercise intensity, *t* ↦ *I*(*t*), is to introduce the oxygen demand, *D*, as an intermediary quantity through coupled ODEs^15^

1 $$\left\{\begin{array}{ll}\dot{D}(t)\;\;\;\,=B\cdot \left(f(I(t))-D(t)\right),\\ \dot{{{{\rm{HR}}}}}(t)\;\,=A\cdot {\left({{{\rm{HR}}}}(t)-{{{{\rm{HR}}}}}_{{\mathrm{min}}}\right)}^{\alpha }\cdot {\left({{{{\rm{HR}}}}}_{{\mathrm{max}}}-{{{\rm{HR}}}}(t)\right)}^{\beta }\cdot \left(D(t)-{{{\rm{HR}}}}(t)\right),\\ {{{\rm{HR}}}}(0)\;={{{{\rm{HR}}}}}_{0},\\ D(0)\;\;\;={D}_{0}.\end{array}\right.$$

In this dynamical system, the function *f* translates the instantaneous activity intensity *I* into the necessary oxygen demand for *I*. The top equation attempts to match the current body oxygen demand *D* with the instantaneous demand *f*(*I*) (also known as the *drive function*). Parameter *B* controls how fast *D* adapts to *f*(*I*). At the same time, the second equation drives the heart rate HR toward the pace required to deliver the demand *D*. Parameter *A* controls how fast the heart can adapt while the terms with HR_min_, HR_max_, and *α*, *β* control how difficult it is to reach the maximal heart rate or to rest down to the minimal heart rate.

To learn the parameters of this ODE, previous studies of such models have limited their data collection to controlled laboratory environments with small samples—typically fewer than ten individuals^14–17^. Workout intensity data *I* was typically the power output or cadence from a stationary bicycle, and the drive function *f*(*I*) was modeled using low-order polynomials^14,15^. Here, we extend the ODE method to large-scale uncontrolled environments and use it to model workout data from wearable devices; we do this by learning some parts of the ODE as neural networks and some parameters of the ODE as user-specific variables. Furthermore, we augment the ODE to incorporate environmental factors, such as temperature and fatigue.

### Modeling heart rate in uncontrolled environments

In uncontrolled environments, accurately measuring the exercise intensity *I* becomes challenging. Instead, wearables rely on sensors like GPS and pedometers to measure proxy variables for activity intensity (such as speed, elevation, and number of steps). However, the relationship between *I* and *f*(*I*) becomes unclear when dealing with such generally noisy measurements. To tackle this challenge, we use a flexible neural network to model and learn the drive function *f*.

Uncontrolled environments might also have external factors that can affect heart rate. For instance, higher temperatures induce a higher oxygen demand^21^. In contrast to controlled environments, workouts in uncontrolled settings may also vary in duration, leading to potential changes in heart rate dynamics over time due to fatigue. Hence, we refine the heart rate equations by modeling how they are affected by the weather *W*, which includes temperature and humidity measurements at the time of workout, and add the effect of fatigue incurred over time *t* during the workout. We parameterize these effects by neural networks *g*(*W*) and *h*(*t*), respectively, and we incorporate them into the demand equation. The term (*f*(*I*(*t*)) − *D*(*t*)) becomes (*f*(*I*(*t*)) ⋅ ***g(W) ⋅ h(t)*** − *D*(*t*)). For instance, *g*(*W*) > 1 indicates an increase in oxygen demand for the weather *W*.

### Learning a personalized large-scale heart rate model

Each individual possesses their own set of personalized parameters (A, B, *α*, *β*, as well as the drive function *f* and HR_min_, HR_max_) that capture their unique heart rate dynamics in response to exercise. Inferring these parameters for each subject and understanding how they might evolve over time can reveal important insight into their health status^17^. However, learning these parameters for every subject and each new workout is computationally expensive. Instead of directly learning a set of parameters for each subject, we assume that an individual’s health state at a given time can be represented by a low-dimensional latent vector $z\in {{\mathbb{R}}}^{\ell }$. Then, we turn each ODE parameter into a function of this health representation. For instance, the parameter *α* becomes *α*(*z*), and *f*(*I*) becomes *f*(*z*, *I*). All these “functions of *z*” are parametrized as neural networks, and our goal will be to learn these health representations.

With these changes, the ODE in Equation (1) becomes

2 $$\left\{\begin{array}{ll}\dot{D}(t)\;\;\;\;=\,B{{{\boldsymbol{(z)}}}}\cdot \left(f({{{\boldsymbol{z,}}}}\,I(t))\cdot {{{\boldsymbol{g(W)}}}}\cdot {{{\boldsymbol{h(t)}}}}-D(t)\right),\\ \dot{{{{\rm{HR}}}}}(t)\;\;=\,A{{{\boldsymbol{(z)}}}}\cdot {\left({{{\rm{HR}}}}(t)-{{{{\rm{HR}}}}}_{{\mathrm{min}}}{{{\boldsymbol{(z)}}}}\right)}^{\alpha {{{\boldsymbol{(z)}}}}}\cdot {\left({{{{\rm{HR}}}}}_{{\mathrm{max}}}{{{\boldsymbol{(z)}}}}-{{{\rm{HR}}}}(t)\right)}^{\beta {{{\boldsymbol{(z)}}}}}\cdot \left(D(t)-{{{\rm{HR}}}}(t)\right),\\ {{{\rm{HR}}}}(0)\;\,=\,{{{{\rm{HR}}}}}_{0}{{{\boldsymbol{(z)}}}}\\ D(0)\;\;\;\,=\,{D}_{0}{{{\boldsymbol{(z)}}}}.\end{array}\right.$$

Fitting our proposed ODE model to large-scale wearable data involves learning both the global shared neural networks and the subject-time specific health representation *z*. In order to efficiently learn the health representation *z*, we finally introduce one last component inspired by deep learning methodologies. We posit that at any time, the subject’s workout history up to this time contains all of the information to characterize *z*. To model the complex interactions that define *z* based on this history, we utilize a convolutional neural network (CNN) encoder architecture. This allows us to learn the subject-specific health representation as a function of their past workout data. Hence, we exchangeably refer to *z* as the *health representation* or the *history embedding*. Figure 2 summarizes the full pipeline of our method: at any given time and for any subject, the model takes the workout history of this subject up to this time and feeds it to an encoder function to obtain a health representation *z*. Subsequently, the representation *z* is transformed into ODE parameters that are used to solve the ODE for new incoming workouts. Training the model end-to-end is done with standard gradient descent to identify the best neural network weights that best predict workout heart rate sequences.

For training and evaluation, we divided the data into a training set and a testing set. The training set comprises the first 80% of workouts for each subject, while the remaining 20% of workouts form the test set and are held out during training. We selected a few hyperparameters using the best training loss. Additional model details and a description of the implementation, neural network hyperparameters, and training procedure using ODE solvers can be found in Supplementary Methods.

## Results

### Heart rate profile forecasting

**Fig. 3:**
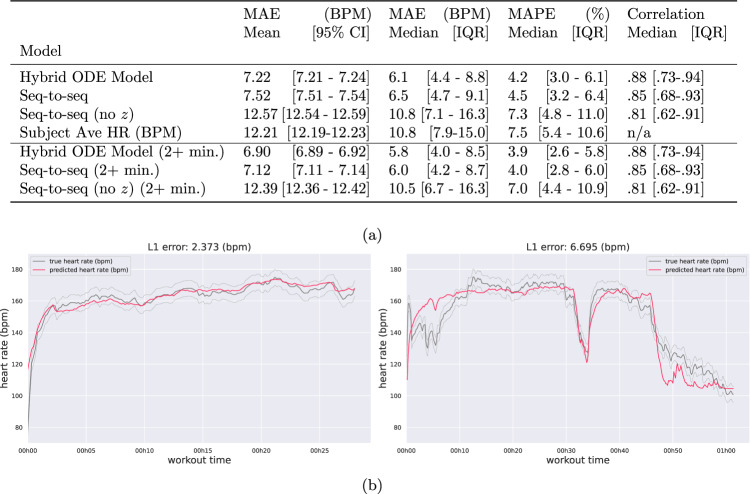
Prediction performance summary. **a** Heart rate reconstruction performance. We compare the average workout mean absolute error (MAE) (and 95 % CI), as well as the median workout MAE and interquartile range (IQR). Additionally, we compare the mean absolute percent error (MAPE) (and IQR) and the prediction sequence correlation (and IQR). We observe the history embedding *z* used in the hybrid ODE model and seq-to-seq baseline improves predictions, and the hybrid ODE model consistently outperforms the strong seq-to-seq baseline. **b** Example HR predictions for two separate workouts. The *x*-axis indicates time since the beginning of the workout and the *y*-axis shows the subject’s instantaneous heart rate (beats per minute). The measured heart rate sequence is in gray, and the predicted sequence is in red. Uncertainty bands about the observation reflect a standard deviation of ± 5 beats per minute in the heart rate measurement. Additional predictions in Supplemental Fig. 7.

The representation *z* estimated using an individual’s workout history can be used to predict the heart rate in future workouts. We measured the accuracy of heart rate prediction on workouts that were held out for each subject. Figure 3 shows two examples comparing the true heart rate to the heart rate estimated using our model (additional predictions in Supplementary Fig. 7). Note that for predicting HR for workout *w* happening at date *T*, our model only uses the workout intensity measures of that sample *I* and the personalized health representation *z*—coming from encoding the previous workouts; i.e., the model does not observe any HR measurements for making predictions.

We compare the prediction performance of our model to seq-to-seq deep models with and without the embedding *z* with similar modeling capacity, as well as a simple heuristic that uses the per-subject mean HR to form the prediction. Figure 3 reports the performance across these baselines, with our model outperforming the context-free (i.e., no *z*) and the strong seq-to-seq baseline. We also measure the performance of our model in estimating the HR after the first 2 min of the workout. Indeed, it is difficult, if not impossible, for a model to predict the heart rate at the beginning of a workout. The initial heart rate depends on the user’s activity prior to the workout, which is unobserved and unpredictable. Conversely, we hypothesize that the heart rate after 2 min can be explained by the user’s activity in the first 2 min that we do observe. Again, we see that the hybrid ODE model outperforms all baselines.

### Oxygen demand inference

One of the key benefits of our hybrid model lies in the interpretability of its latent variables. Supplementary Fig. 8 depicts the inferred demand curve *D* for a set of random workouts. We observe that the demand is highly correlated with HR, but typically at a lag—changes in HR tend to follow changes in demand, and the speed of those changes is described by the ODE parameters.

### Calories burned estimation

The number of calories burned during exercise can be approximated using heart rate measurements during the workout with a linear formula^22^. This, which is only a first-order approximation often augmented with other movement measurements, is useful for planning workouts based on calorie burn goals and even more useful in cases where individuals are not wearing a wearable device that records heart rate. Our method can reliably estimate the number of calories burned with a 5% relative error (the same relative error as the heart rate reconstruction), only using workout metrics that can be measured using a smartphone.

### Heart rate zone prediction

**Fig. 4:**
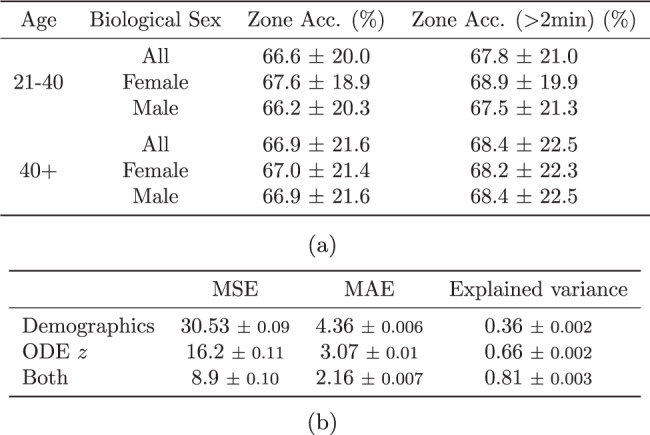
Heart rate zone and VO_2_ max prediction results summary. **a** Predictive performance of heart rate zones (mean and standard deviation). The most frequent zone occurred 38% of the time on average, which corresponds to the accuracy the best possible baseline predictor can achieve. **b** VO_2_ max prediction performance in mean squared error (MSE) and mean absolute error (MAE) (mean and standard error).

Exercise heart rate zones are the percentage of an individual’s age-related maximum heart rate reached throughout the course of exercise, where maximum heart rate is derived using the common 220 bpm − age heuristic^23^ (distinct from the ODE model parameter). Using our physiological model, we can predict heart rate zones using the workout data. This can help individuals plan personalized exercise routines to achieve their fitness goals more effectively. We define six zones (% intervals [0, 50, 60, 70, 80, 90, 100]) of maximum heart rate, and Fig. 4a shows the performance of our method in predicting the HR zone for the whole population, as well as different subgroups of the population. We find that the model can predict heart rate zones with an accuracy of around 67%. To provide a comparison, we computed the marginal distribution of true heart rate zones across all workouts and found that the most frequent zone ([80,90]) occurs about 38% of the time. So the best possible constant predictor would be correct about 38% of the time—significantly lower than the accuracy achieved by our model.

### Quantifying the impact of the weather on heart rate

**Fig. 5:**
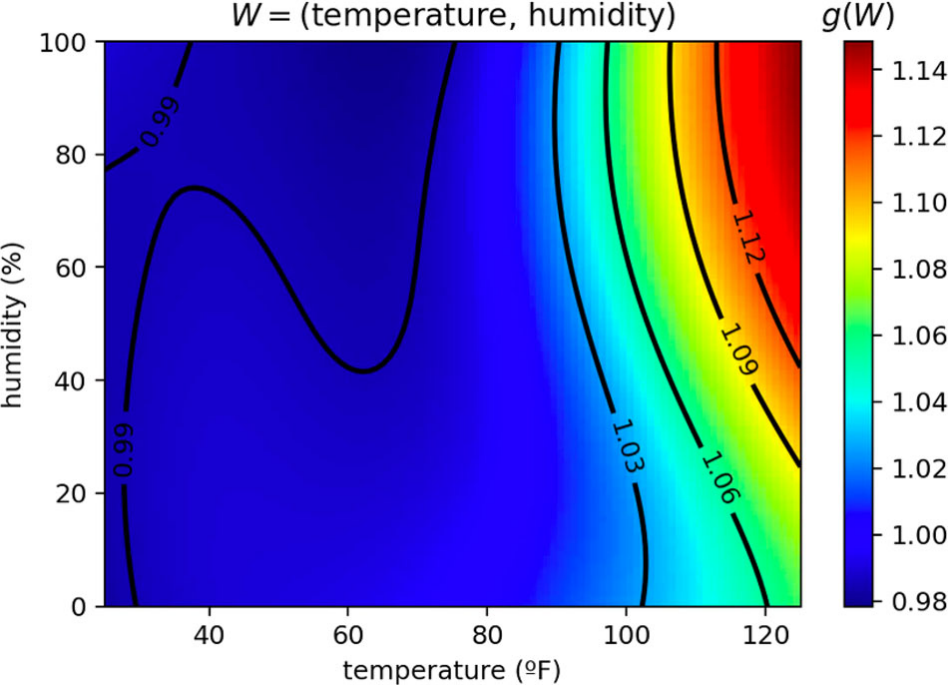
Impact of weather. Impact of weather temperature and humidity on the body’s oxygen demand *g*(*W*), as modeled by the differential equation. As temperature and humidity rise, we see an increase in oxygen demand, amounting to a 3-6% increase near 100 degrees (or about 4.5–9 BPM at 150 BPM effort).

Leveraging the interpretability of our ODE model, we analyze the learned neural network *g* and quantify the relative effect of weather on the body’s oxygen demand. This constitutes one of the largest studies of this kind (over 270,000 workouts). The neural network *g* is a global function shared between all subjects and workouts. Figure 5 shows an increase in body oxygen demand by up to 10% in high temperatures and humidity. Moreover, we found that personalizing *g* as *W* ↦ *g*(*z*, *W*) in the same way that the personalized exercise intensity function *f* is parameterized by the representation *z* did not result in significant improvements in heart rate predictions. We kept a shared *g* for simplicity and interpretability.

### Learning about cardiorespiratory health

To check that our representations summarize information about cardiorespiratory health, we use a summary of cardio fitness, VO_2_ max, estimated by wearable devices. VO_2_ max is the maximum rate of oxygen the body can consume during exercise, normalized by body mass. While a cardiopulmonary exercise test (CPET) is the gold standard for measuring VO_2_ max, such tests can be prohibitively expensive and even infeasible for certain populations. Instead, we can approximate VO_2_ max from sub-maximal exercise bouts using measurements collected in our study^24^, including heart rate, GPS, and user informaiton^25^.

Using the health representations *z*, we predict the estimated VO_2_ max with a simple linear regression model and achieve a mean absolute error of ± 2.16 mL/(kg ⋅ min), which is about 5% of the average VO_2_ max in the data (42.5 mL/(kg ⋅ min)). Figure 4b reports the performance of a linear regression model on the ODE representations only, on demographics only, or on both. The demographics include subject height, weight, biological sex, and age. Supplementary Fig. 11 shows a 2D projection of the health representation for different workouts where we can see the separation of higher and lower values of VO_2_ max. We also examined the association between the *z* representations and subject age and body mass index (BMI). With a linear regression model, we find that the *z* representations explain 33% ( ± 0.3 %) of the variance in age and 16% ( ± 0.7%) of the variance of BMI in our cohort.

### Interpreting hybrid ODE model inferences

We also investigate the relationship between the inferred ODE parameters (*A*, *B*, *α*, and *β*) and age, sex, and fitness. Supplementary Fig. 9 illustrates the variation of these four parameters as a function of age (on the *x*-axis), stratified by VO_2_ max tertiles. As previously mentioned, a higher value of *α* suggests that individuals can more easily approach their resting heart rate. We observe that the highest VO_2_ max tertile exhibits a significantly higher inferred *α*, although the gap disappears as the cohort ages. Similarly, a higher value of *β* indicates that individuals can more readily reach their maximum heart rate. Among the youngest cohort, we observe that the fittest group can reach the inferred maximum heart rate more quickly. The parameter *A* characterizes the overall sensitivity of heart rate changes. In the younger age range, the least fit (lowest VO_2_ max cohort) displays a significantly higher average *A* value, which diminishes as age increases. Lastly, parameter *B* signifies the sensitivity of the demand sequence *D* to changes in exercise intensity. We note that this value exhibits less variability across ages and VO_2_ max strata.

## Discussion

The increasing availability of wearable devices is enabling individuals to track their health and fitness. We developed a method that predicts heart rate response to workout intensity using data from a wearable device. We learn representations that summarize the dynamics of the HR response, by combining machine learning techniques with an expert model from the exercise physiology literature. All results are derived from one of the largest studies in the general population (outside of the lab), illustrating the power of wearables in real, everyday scenarios.

We show that this hybrid model can accurately predict heart rate sequences for new workouts given a user-specific history of recent workouts. Beyond heart rate predictions, we show that representations from this algorithm can serve as a measure of cardiorespiratory fitness, which can help track fitness levels over time and aid personalized workout planning. Additionally, we show evidence of the effect of weather on heart rate demand across the study population.

Methodologically, we demonstrate how machine learning techniques can be used to translate an expert model—originally developed for a controlled setting—to noisy, real-world signals collected by wearables throughout a subject’s daily life. We use ML techniques to both augment the model (e.g., using a neural network to model the intensity-demand function) and to scale the algorithm to a large dataset of users and workouts (e.g., via learned subject-specific history embeddings).

There remain multiple avenues for future work. The first is further developing applications of this model for planning new workouts and tracking fitness changes over time. While predictions of heart rate response describe user fitness, it remains an open question how to translate insights from such a model to improvements in fitness over time. With respect to behavior change, the studied effects of incentivizing exercise adherence remain unclear^26,27^, and the extent to which personalized insights may help is unknown.

Additionally, we aim to better understand how these learned representations are associated with or predict changes in cardiovascular function and adverse cardiovascular outcomes. Cardiovascular health is strongly associated with exercise and fitness level^28^, and physical activity can be a preventative activity and prognostic indicator for heart failure^29^. And while we focus on running workouts, lower-intensity walking workouts may also carry rich information about an individual’s cardiovascular health—adaptations of our approach could be applied to walking workouts (or both walking and running workouts).

Lastly, this work proposes a new methodology to combine expert models of physiology alongside machine learning components. While we observe that this expert model can provide an inductive bias that makes predictions more accurate, it remains to be studied how accurately physiologically interpretable parameters can be identified and inferred from wearables data. Furthermore, more complex models of heart rate—e.g., directly parameterizing VO_2_ max or exercise thresholds—remain to be studied within this hybrid framework.

A limitation of the study design is that participants must have access to an iPhone with the Research app and an Apple Watch to be eligible for participation. Furthermore, we select individuals who have running workouts to train and evaluate our models. Further study is required to understand if our approach is accurate under a less active population—such selection bias may confound our interpretation of inferred physiological parameters. Another limitation is that this study focuses only on outdoor running workouts. In theory, this approach could be extended to other activities, such as cycling or indoor runs, provided the analogous workout intensity is sufficiently rich. Additionally, our study is limited to questions of cardiorespiratory fitness, and not subsequent health outcomes. Follow-up on more detailed longitudinal data is necessary to establish a strong link between representations learned by our algorithm and subsequent health-related events.

### Supplementary information

Supplemental Material

## Data Availability

Due to privacy and consent considerations, data from the AHMS cannot be shared. We do provide a detailed algorithmic implementation of our approach, and readers interested in the tools can contact the authors for further support.

## Code availability

The code used to run our model will be hosted at https://github.com/apple/ml-heart-rate-models upon acceptance. This repository includes methods for preprocessing data, fitting models, and making predictions.
