# Supplementary material for: Modeling personalized heart rate response to exercise and environmental factors with wearables data
Source: NPJ Digit Med. 2023 Nov 15;6:207. doi: 10.1038/s41746-023-00926-4 (PMC10651837; doi:10.1038/s41746-023-00926-4)
Supplement: Supplementary file 1 — Supplemental Material [file 41746_2023_926_MOESM1_ESM.pdf]

Supplementary Materials: *Modeling Personalized Heart Rate Response to Exercise and Environmental Factors with Wearables Data*

Achille Nazaret, Sana Tonekaboni, Greg Darnell, Shirley Ren, Guillermo Sapiro, and Andrew C. Miller

Supplementary Methods

Personalized large-scale heart rate model. The generative model associated with the proposed ODE model is the following:

- For each subject i and workout w, write the time of the workout as T_w, and
  - Draw the health representation of subject i at time T_w:

    $$z_{i,T_w} \sim \mathcal{N}(0, I_\ell);$$

    (this is equivalent to L2 regularization of the health representation)
  - Compute ODE parameters from z_{i,T_w} and neural networks: $\alpha(z_{i,T_w})$, $\beta(z_{i,T_w})$, $A(z_{i,T_w})$, $B(z_{i,T_w})$, $\mathrm{HR}_{min}(z_{i,T_w})$, $\mathrm{HR}_{max}(z_{i,T_w})$;
  - Solve the ODE during the workout w with the computed parameters and the available exercise intensity $t \mapsto I^{(w)}(t)$, environmental effect $g(W)$, and workout time effect $h(T)$. Obtain the expected heart rate sequence during workout $t \mapsto \mathrm{HR}^{(w)}(t)$;
  - For each time t, draw the observed heart rates measured by the wearable device:

    $$\widehat{\mathrm{HR}}^{(w)}(t) \sim \mathcal{N}\Big(\mathrm{HR}^{(w)}(t), \sigma = 5\Big).$$

    (this is equivalent to computing the squared loss between the reconstructed heart rate and the observed heart rate, with a standard deviation of 5).

For simplicity, we fixed the observation standard deviation to ±5 beats per minute, which is the average estimation error of wearable heart rate sensors [7]. A graphical representation of this full model is depicted in Supplementary Figure 6

ODE parameter networks. The network that generates ODE parameters from embeddings is a multi-layer perceptron with two hidden layers, of size 32 and 8, with a softplus nonlinearity. The output of this network is constrained to produce values in a reasonable range for each of the physiological parameters using a scaled logistic function. ODE parameter ranges are

- $A \in (-3, 5)$,
- $B \in (-3, 5)$,
- $\alpha \in (0.1, 3.0)$,
- $\beta \in (0.1, 3.0)$.

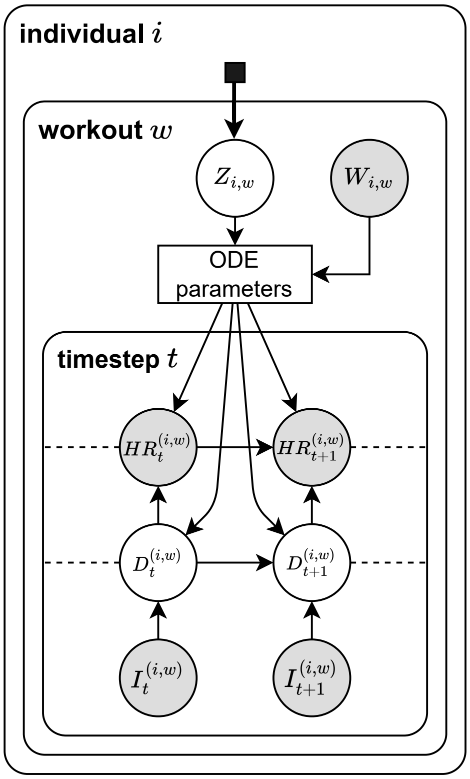

Figure 6: Graphical model representation of the heart rate modeling ODE. The latent variable $Z_{i,w}$ summarizes the individual's fitness level. This representation, along with the observed measurements of a new workout, approximates the personalized ODE parameters that model the heart rate response.

The drive function $I \mapsto f(I)$. The oxygen demand neural network is a multi-layer perceptron with two hidden layers $(128, 64)$ that maps the four-dimensional workout intensity vector (i.e., horizontal speed, vertical speed, cadence, and GPS speed), concatenated with the history embedding z to produce a subject-specific demand response.

The weather network $W \mapsto g(W)$. The function that describes the response to weather, $g(W)$, maps a 2-dimensional weather summary to a 1-dimensional rescaling effect and is instantiated as a multi-layer perceptron with two hidden layers (32, 16), with total effect constrained to be between 0.5 and 1.5.

The fatigue network $t \mapsto h(t)$. The function that models the effect of workout duration, $h(t)$, maps a 1-dimensional time variable to a 1-dimensional rescaling effect and is also instantiated as a multi-layer perceptron with two hidden layers, with total effect constrained to be between 0.5 and 1.5.

Encoder convolutional neural network. To form the history that will be embedded into a health representation by the encoder for a given time T and subject i, we concatenate the data from all workouts of subject i that preceded time T. The heart rate measurements and the exercise intensity of these workouts, as well as the time elapsed between these past workouts and the target time T, are concatenated into a multivariate time series. The times between past workouts and the target time T are log-transformed for numerical stability, and the history is limited to the last 1000 most recent measurements for scalability.

Following [13], we embed the workout history using a causal convolutional neural network. We use adaptive average pooling to accept variable-length history. The results presented use a 64-dimensional hidden layer with a 32-dimensional output embedding and a kernel width of 6.

Implementing and training the model. The model is implemented in Python using the PyTorch library. The ODE is solved using the Fourth Order Runge-Kutta method from the Python library `TorchDiffEq` [10] in such a way that yields an HR solution that is differentiable against its input parameters (in order to use gradient descent). As noted in the generative model above, the objective function that we minimize comprises the squared loss between the reconstructed heart rate and the observed heart and the regularization of the neural networks' weights by an L2 penalty. In practice, we subsample batches or portions of workouts and use stochastic gradient descent with the Adam optimizer. The gradient updates simultaneously learn the representation encoder and all the ODE internal neural network parameters. This is detailed in Algorithm 1.

Algorithm 1 Training loop.

Require: Training data, number of epochs $e = 100$, learning rate $\alpha = 0.001$, L2 regularization strength $\gamma = 1$.

for each epoch e **do**
  for each row r of data **do** ▷ In practice this loop is vectorized over minibatches.
    $(\text{HR}, I, t, W, history) \leftarrow r$ ▷ Heart rates, Intensities, times, Weather
    $z \leftarrow encoder(history)$
    $\widehat{\text{HR}} \leftarrow ODE(A(z), B(z), \text{HR}_{min}(z), \text{HR}_{max}(z), \alpha(z), \beta(z), \text{HR}_0(z), D_0(z), f(I, z), g(W), h(t))$
    $L \leftarrow (\frac{\text{HR}-\widehat{\text{HR}}}{5})^2$
    $L2 \leftarrow$ L2 regularization over all neural networks' parameters
    Take a gradient step on $L + \gamma L2$, with Adam(learning rate= α)
  end for
end for

Preparation of the data. The dataset is reorganized before training so the data can be accessed quickly during training. We ensure that the full history of the users' activity preceding each workout is readily accessible. In addition, we also cut workouts into smaller workouts of length $S = 64$ to increase the training speed. This is detailed in Algorithm 2.

Algorithm 2 Dataset preparation

Require: Workouts data, length S of workout portions subsampled for training.

```
dataset ← [ ]
for each user i do
    history ← [ ]
    for each workout w of user i do          ▷ The workouts must be sorted by increasing date.
        T ← date and time of workout w
        L ← length of w                      ▷ The duration of w is 10 · L seconds
        HR ← heart-rate of w                 ▷ Vector of shape [L, 1]
        I ← exercise intensities of w        ▷ Vector of shape [L, 4]
        t ← [0, 10, ..., 10 · L]             ▷ Seconds elapsed at each measure of HR or I.
        W ← weather during w
        h ← history.copy()
        h[:, −1] ← log(T − h[:, −1])         ▷ Duration between past and current workout.
        if training data then                ▷ Workouts are subsampled into smaller portions for training.
            s ← 0
            while s < L do
                d ← (HR[s : s + S], I[s : s + S], t[s : s + S], W, history)
                dataset.append(d)
                s ← s + S
            end while
        else                                 ▷ Workouts of the test set are not subsampled.
            d ← (HR, I, t, W, history)
            dataset.append(d)
        end if
        history.append([HR, I, T])           ▷ [HR, I, T + t] has shape [L, 6]
    end for
end for
```

Supplementary Figures

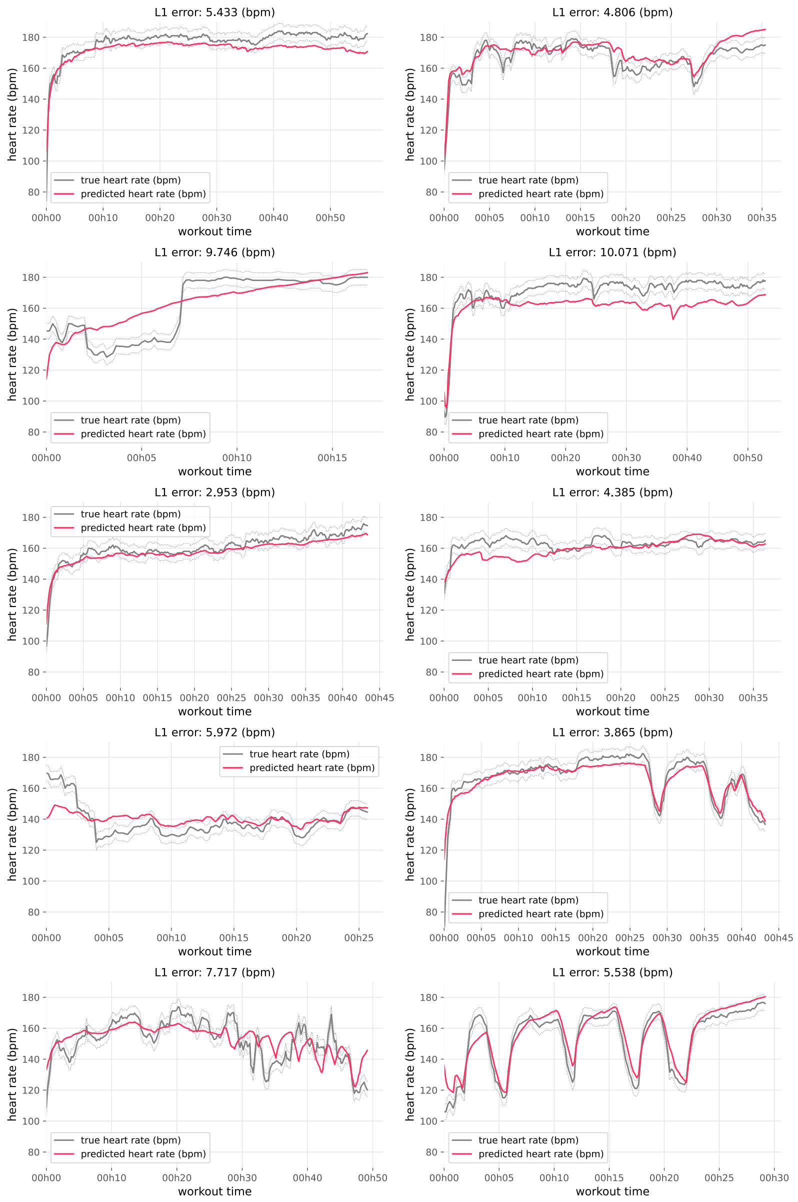

Figure 7: A random sampling of ten workout predictions.

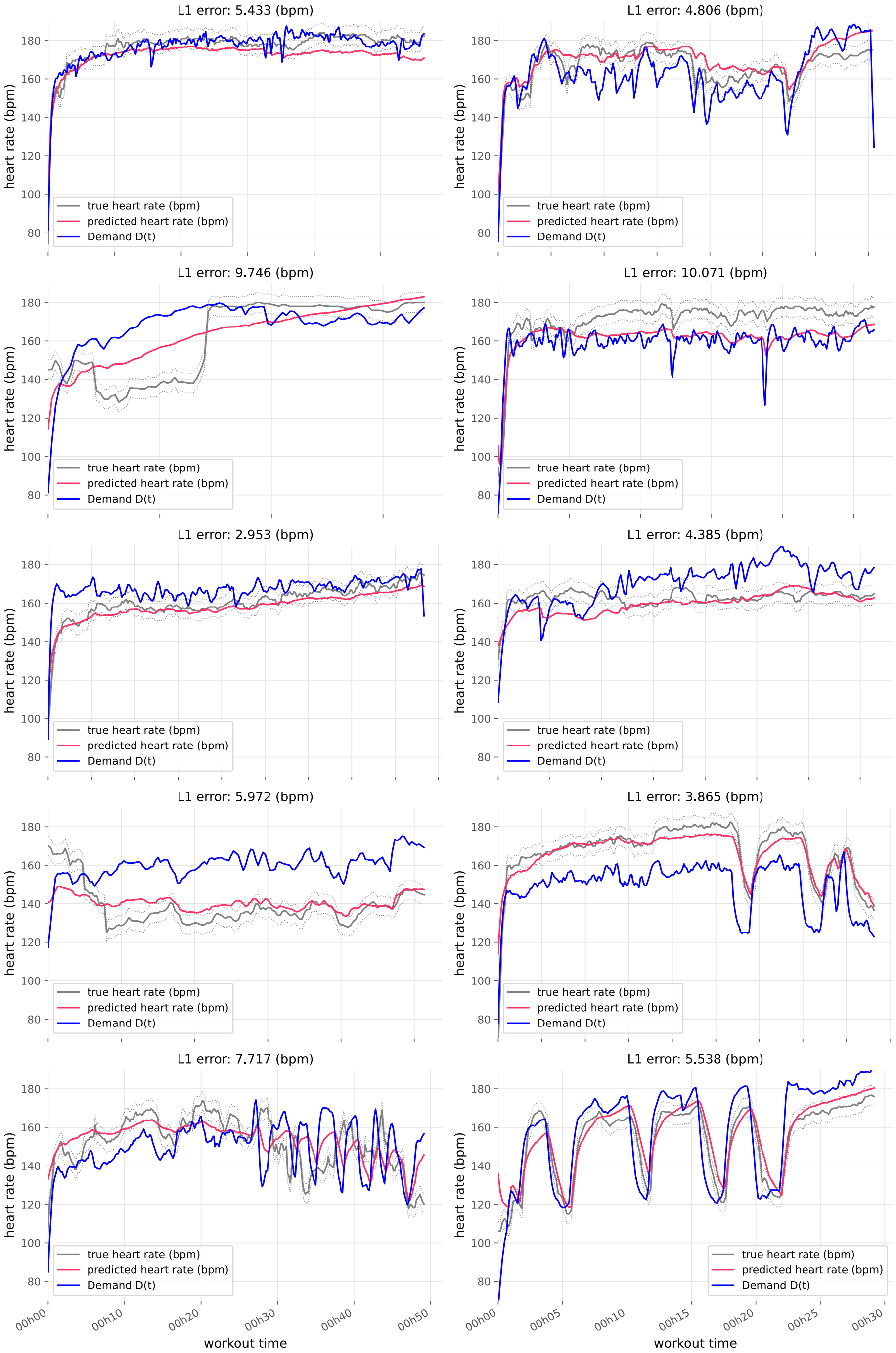

Figure 8: Additional HR predictions, also visualizing the intermediate demand sequence.

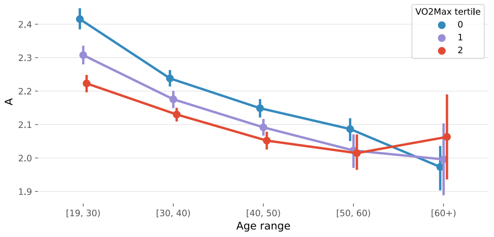

(a) A

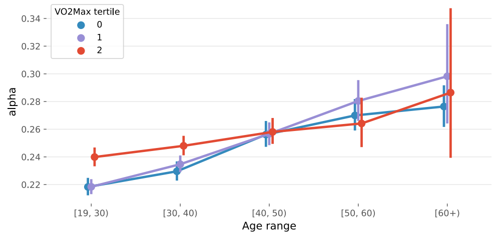

(b) α

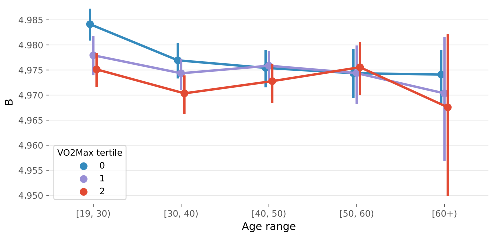

(c) B

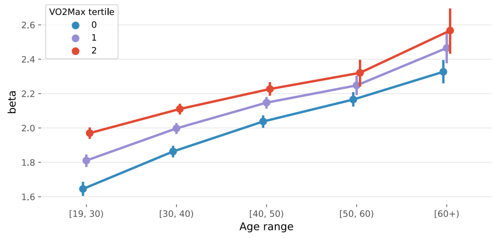

(d) β

Figure 9: Inferred physiological parameters by age and fitness level (VO_2 max tertiles). We see a large separation between VO_2 max groups for α, β, and A, with the difference tending to shrink for the older cohort. Error bars correspond to 95% intervals using 1,000 bootstrap samples.

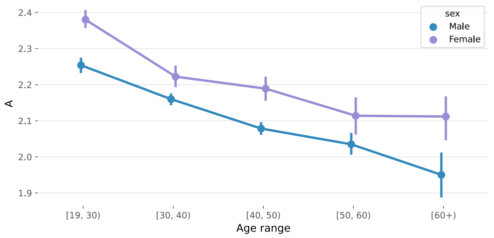

(a) A

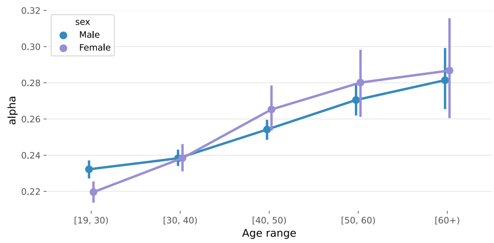

(b) α

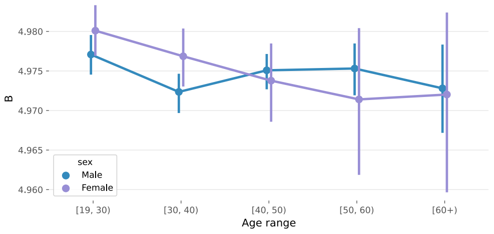

(c) B

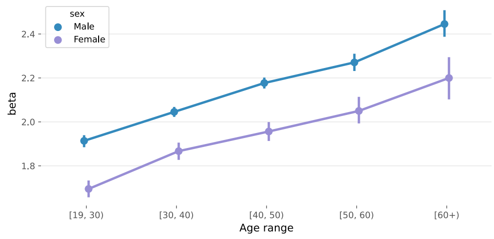

(d) β

Figure 10: Inferred physiological parameters by age and sex. We see a large separation between sex for the A and β parameters. Error bars correspond to 95% intervals using 1,000 bootstrap samples.

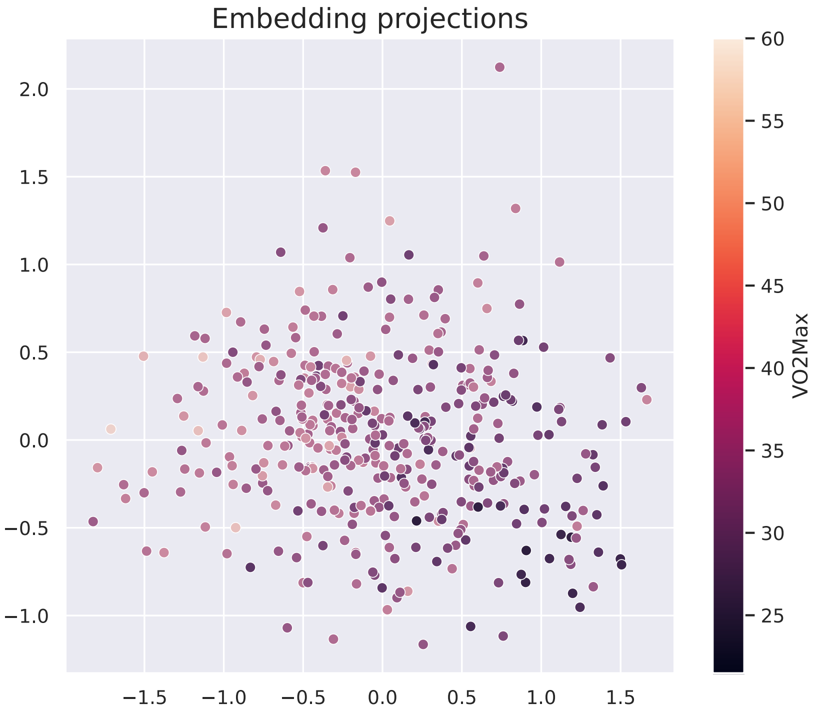

Figure 11: Projection of the first two principal components of the per-workout Z representations, colored by VO_2 max (darker is lower). Visually, we can see lower (darker) VO_2 max points more densely packed in the lower right hand corner — a pattern that is corroborated and quantified by the predictive model results in Figure 4(b).
